# Preclinical development of G1T38: A novel, potent and selective inhibitor of cyclin dependent kinases 4/6 for use as an oral antineoplastic in patients with CDK4/6 sensitive tumors

**DOI:** 10.18632/oncotarget.16216

**Published:** 2017-03-15

**Authors:** John E. Bisi, Jessica A. Sorrentino, Jamie L. Jordan, David D. Darr, Patrick J. Roberts, Francis X. Tavares, Jay C. Strum

**Affiliations:** ^1^ G1 Therapeutics, Preclinical Research and Development, Research Triangle Park, Durham, North Carolina, USA; ^2^ University of North Carolina, MP1U, Chapel Hill, North Carolina, USA; ^3^ ChemoGenics BioPharma, Department of Chemistry Research Triangle Park, Durham, North Carolina, USA

**Keywords:** G1T38, CDK4/6, cyclin dependent kinase inhibitor, NSCLC, ER^+^ breast cancer

## Abstract

Inhibition of the p16^INK4a^/cyclin D/CDK4/6/RB pathway is an effective therapeutic strategy for the treatment of estrogen receptor positive (ER^+^) breast cancer. Although efficacious, current treatment regimens require a dosing holiday due to severe neutropenia potentially leading to an increased risk of infections, as well as tumor regrowth and emergence of drug resistance. Therefore, a next generation CDK4/6 inhibitor that can inhibit proliferation of CDK4/6-dependent tumors while minimizing neutropenia could reduce both the need for treatment holidays and the risk of inducing drug resistance.

Here, we describe the preclinical characterization and development of G1T38; a novel, potent, selective, and orally bioavailable CDK4/6 inhibitor. *In vitro*, G1T38 decreased RB1 (RB) phosphorylation, caused a precise G1 arrest, and inhibited cell proliferation in a variety of CDK4/6-dependent tumorigenic cell lines including breast, melanoma, leukemia, and lymphoma cells. *In vivo*, G1T38 treatment led to equivalent or improved tumor efficacy compared to the first-in-class CDK4/6 inhibitor, palbociclib, in an ER^+^ breast cancer xenograft model. Furthermore, G1T38 accumulated in mouse xenograft tumors but not plasma, resulting in less inhibition of mouse myeloid progenitors than after palbociclib treatment. In larger mammals, this difference in pharmacokinetics allowed for 28 day continuous dosing of G1T38 in beagle dogs without producing severe neutropenia. These data demonstrate G1T38 has unique pharmacokinetic and pharmacodynamic properties, which result in high efficacy against CDK4/6 dependent tumors while minimizing the undesirable on-target bone marrow activity, thus potentially allowing G1T38 to be used as a continuous, daily oral antineoplastic agent.

## INTRODUCTION

In normal cells, the cyclin D/CDK4/6/p16^INK4a^/RB pathway allows for the orderly control of cell cycle progression for cell growth and proliferation. While the cyclin D/CDK4/6/p16^INK4a^/RB pathway is frequently disrupted in cancer, the majority of human neoplasms maintain functional RB but have aberrations that increase the activity of CDK4/6, which hyper-phosphorylates RB and allows cell proliferation [1, 2]. As such, CDK4/6 appears to be a key enzyme necessary for the proliferation of human cancers that have functional RB.

Cell cycle progression begins with the commitment to transition from the G1 phase to the S phase; this restriction point is regulated through RB. In the absence of a growth signal, RB inhibits the activation of genes required for S phase transition [1, 2]. Growth signals activate CDK4/6, which phosphorylates and deactivates RB, thus activating S phase-specific genes that stimulate cell cycle progression from the G1 phase to the S phase [3]. Further, inhibiting CDK4/6 activity has the potential to be therapeutically beneficial in a wide variety of tumors with functional RB.

There are numerous ways in which cancers with functional RB enhance CDK4/6 activity allowing for deregulated proliferation. One of the most common events is the inactivation of p16^INK4a^, a tumor suppressor that inhibits CDK4/6 activity. Inactivation of p16^INK4a^ may occur via mutations, deletion, or epigenetic silencing, which is observed in a significant portion of non-small cell lung cancer, melanoma, pancreatic cancer, and mesothelioma [4–8].

Moreover, a specific mutation of the CDK4 gene (CDKR24C) that confers resistance to p16 ^INK4a^ binding has been shown to play a causal role in rare cases of familial melanoma, suggesting that unchecked CDK4 activity is a key event in these cancers [9]. Translocation or amplification of D-type cyclin genes that lead to increased CDK4/6 activity are found in the majority of mantle cell lymphomas and in many cases of multiple myeloma [10, 11]. Amplification of cyclin D1 and overexpression of the protein have also been reported in approximately 50% of squamous cell esophageal cancers and in 20% to 30% of breast cancers [12–14]. Specifically, the profiling of a breast cancer cell line panel with palbociclib, a selective CDK4/6 inhibitor, demonstrated that CDK4/6 inhibitors are exquisitely effective at inhibiting the growth of ER^+^ cells [15].

Additionally, many of the receptor-mediated growth pathways that are activated in human cancers increase D-type-cyclin transcription and expression to drive cellular proliferation [16, 17]. Other activating aberrations of mitogen pathways such as V600E B-Raf in the mitogen activated protein kinase pathway and phosphatase and tensin homolog deletions in the phosphatidylinositol-3,4,5-trisphosphate 3-phosphatase (PTEN) pathway also increase D-type-cyclins to achieve unchecked proliferation [18, 19]. Furthermore, the genes encoding CDK4 and CDK6 are amplified in a subset of human neoplasms. The CDK4 gene is amplified in 100% of liposarcomas, while CDK6 is frequently amplified in T-lymphoblastic lymphoma and acute lymphoblastic leukemia [20, 21].

Finally, there is increasing evidence the E2F-induced cyclin/CDK hyperactivity in prostate cancer is an ideal target for CDK4/6 inhibition [22]. Androgen receptor dependent upregulation of D-type-cyclins leading to activation of the cyclin D1/CDK4/6 complex and proliferation in prostate cancer cells suggests that targeting this axis may be effective in castration resistant prostate cancer (CRPC). Utilizing CRPC models, G1T38 was observed to have equivalent anti-tumor activity to the standard of care, docetaxel. [Stice JP, Wardell SE, Norris, JD et al., in press].

The abundance of mechanisms by which transformed cells can deregulate CDK4/6 activity suggests this enzyme is crucial for cancer pathogenesis and inhibiting CDK4/6 activity has the potential to be therapeutically beneficial.

In February 2015, the first selective CDK4/6 inhibitor, palbociclib, was approved by the Food and Drug Administration (FDA) to be used in combination with letrozole as initial endocrine-based therapy for the treatment of postmenopausal women with ER^+^, human epidermal growth factor receptor 2 (HER2) negative advanced or metastatic breast cancer. In February 2016, palbociclib was approved in combination with fulvestrant for the treatment of women with ER^+^, HER2^−^ advanced or metastatic breast cancer with disease progression following endocrine therapy. Although palbociclib is efficacious, daily treatment causes severe neutropenia, which necessitates a 21-day on/7-day off schedule to allow for recovery of neutrophil counts [23, 24]. The palbociclib-induced neutropenia may lead to an increased risk of infections and the treatment holiday may lead to the potential for tumor regrowth and emergence of drug resistance. This CDK4/6 dependent on-target effect on HSPC proliferation has been described previously [25, 26], whereby early hematopoietic progenitor cells in the presence of palbociclib are maintained in a G1 arrested state until released. Continuous exposure decreases myelogenesis leading to myelosuppression, which is reversible. Thus, it may be advantageous to develop a next generation CDK4/6 inhibitor that can inhibit cell proliferation in CDK4/6-dependent tumors while minimizing effects on the bone marrow, thereby reducing the need for treatment holidays and decreasing the risk of inducing drug resistance.

Here we describe the preclinical development of G1T38: a novel, oral, potent and selective inhibitor of CDK4/6 that inhibits RB phosphorylation *in vitro* and *in vivo*. G1T38 is shown to possess unique pharmacokinetic and pharmacodynamic properties, which may enable a continuous dosing regimen. By minimizing undesirable on-target myelosuppression, G1T38 may obviate the need for a treatment holiday. G1T38 has recently been tested in a phase 1a, healthy volunteer pharmacokinetics and safety study (G1T38-01, NCT02821624). We are currently evaluating G1T38 (in combination with Faslodex) in a Phase 1b/2a trial in ER+, HER2- breast cancer patients (G1T38-02, NCT02983071).

## RESULTS

### Identification of G1T38

**Figure 1:**
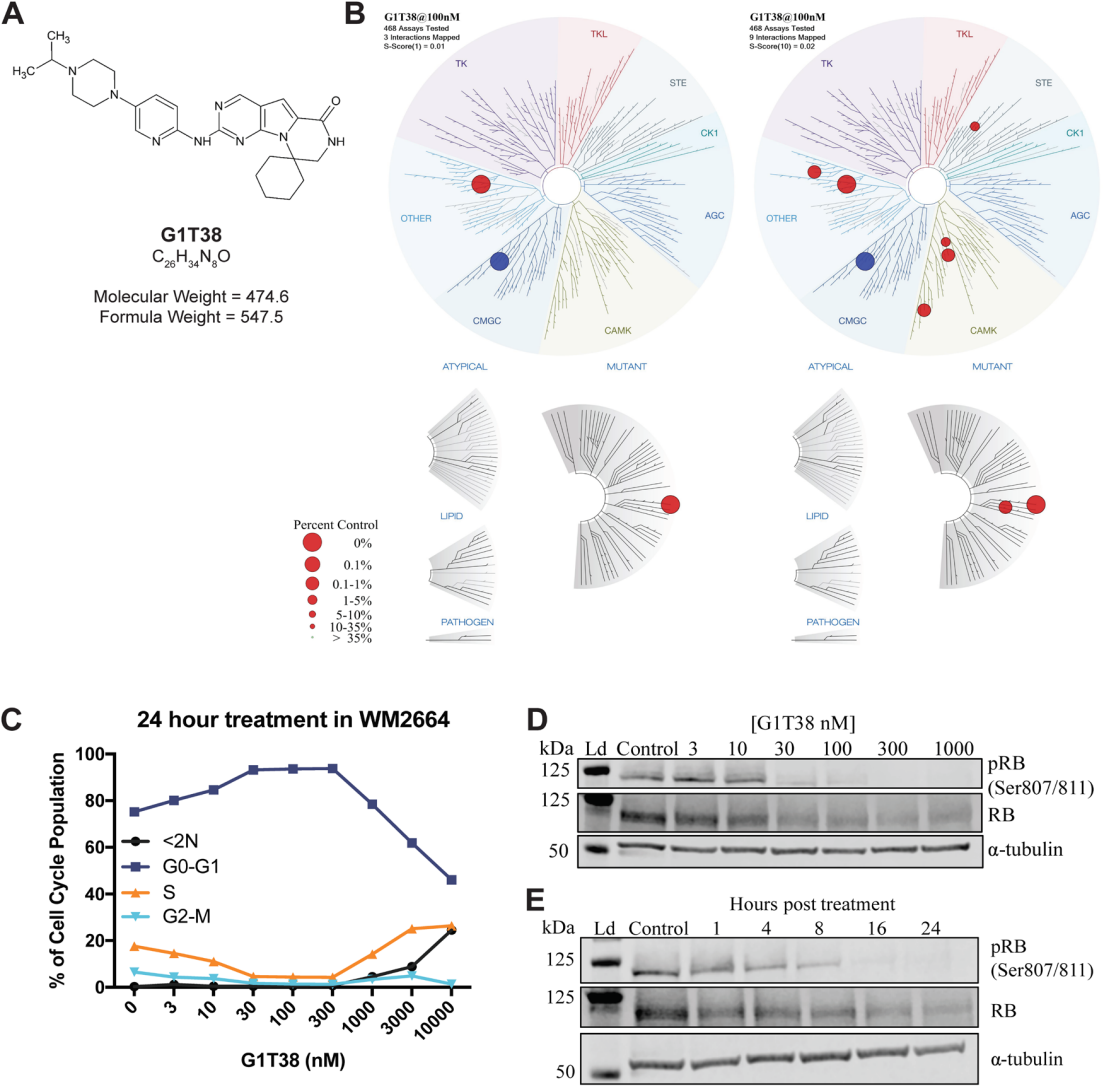
Chemical structure, kinome specificity and biochemical properties of G1T38 **(A)** Structure, molecular formula, and formula weight of G1T38. **(B)** Kinome binding specificity of G1T38 was measured by site- directed competition-binding assays (S-Score= 1, left, S-Score= 10, right). Blue circle represents CDK4/cyclin D3 **(C)** WM2664 cells show a dose-dependent G1 arrest with corresponding percent decrease in the number of cells in S-phase. **(D)** WM2664 cells treated with G1T38 for 24 hours show a dose-dependent decrease in RB phosphorylation with complete inhibition of RB phosphorylation at a 300 nM exposure. **(E)** G1T38 (300 nM) time-dependent inhibition of RB phosphorylation demonstrating complete inhibition 24 hours post treatment. Total RB was assessed as a comparator to relative effect on RB phosphorylation and α-tubulin was used as the loading control.

We have previously reported on a novel kinase inhibitor scaffold and the discovery of trilaciclib (G1T28), an IV, short-acting, potent and selective inhibitor of CDK4/6 currently in clinical development to preserve HSPC and immune system function during chemotherapy [27]. Further development of the tricyclic lactam scaffold has produced a number of oral, potent and selective CDK4/6 inhibitors with excellent drug like properties. G1T38, 2′-((5-(4-isopropylpiperazin-1-yl)pyridin-2-yl)amino)-7′, 8′dihydro-6′Hspiro[cyclohexane1,9′ pyrazino[1′,2′:1,5]pyrrolo[2,3-d]pyrimidin]-6′-one di-hydrochloride (Figure 1A), was selected as a candidate oral antineoplastic agent with ideal physicochemical and pharmacokinetic/pharmacodynamic (PK/PD) properties. G1T38 is highly potent and selective for CDK4/cyclin D1 and CDK6/cyclin D3 over CDK1, CDK2, CDK5 and CDK7 and their respective binding partners (Table 1).

**Table 1:** *In vitro* biochemical activity of G1T38

	CDK4/Cyclin D1	CDK6/Cyclin D3	CDK9/Cyclin T	CDK5/p35	CDK5/p25	CDK2/Cyclin A	CDK1/Cyclin B1	CDK7/Cyclin H/ Mat1	CDK2/Cyclin E
Mean (μM)	.001	.002	.028	0.832	1.2	1.5	2.4	2.4	3.6

Biochemical profiling of G1T38 in Nanosyn, Inc. (Santa Clara, CA) kinase screen for CDKs. The assays were completed using microfluidic kinase detection technology (Caliper Assay Platform). The compounds were tested in 12-point dose response format in singlicate at Km for ATP. Results are displayed as micromolar concentrations for IC_50_ curves against putative target.

To determine kinase selectivity beyond the cyclin-CDK family, we completed a screen of 468 protein kinases (KINOMEscan, DiscoveRx) at 100 nmol/ L and 1 μmol/L, which represents profiling at 100 and 1000 times the biochemical IC_50_ of G1T38. Results in Figure 1B show a high degree of selectivity at 100 nmol/L at an s-score of 1 (Figure 1B, left) and an s-score of 10 (Figure 1B, right). Protein kinases with off-target activity showing inhibition greater than 90% at 100 nmol/L were followed up for Kd determination (Supplementary Table 1). Within the CDK family, G1T38 is least selective against CDK9/cyclin T; ~30 fold between CDK4/cyclin D1 and CDK9/cyclin T at the biochemical IC_50_. The phosphorylation of RNA polymerase II (RPB1) CTD (SER2) was measured via immunoblot to determine whether G1T38 has a biological inhibitory effect on CDK9/cyclin T function. Results show that there is no loss of RPB1 CTD (SER2) phosphorylation in the presence of G1T38 (Supplementary Figure 1). Other targets demonstrating low Kd IC_50_ values include ULK2, an inducer of autophagy and NEK10, which is associated with G2/M arrest in response to UV irradiation. The effect of G1T38 on NEK10 and ULK2 is an area of interest being further assessed as part of the investigation of this molecule.

### G1T38 produces a precise G1 arrest of the cell cycle and a subsequent decrease in proliferation of CDK4/6-dependent cell lines

Next, we assessed the ability of G1T38 to elicit a G1 arrest in normal and tumorigenic CDK4/6-dependent (HS68, WM2664, respectively) and -independent tumorigenic (A2058) cell lines. G1T38 produced a robust and sustained G1 arrest in CDK4/6 dependent cells with an EC_50_ of ~20 nmol/L (Figure 1C). In CDK4/6 independent cells, the compound had no effect on the percentage of S-phase cells (as shown by arrow in Supplementary Figure 1). A dose dependent increase of cells in the G1 phase of the cell cycle was observed when CDK4/6 dependent WM2664 cells were treated with G1T38 for 24 hours (Figure 1C). This arrest was maintained through 300 nM, more than 300x the biochemical IC_50_. Additionally, both CDK4/6-dependent cell lines (HS68 and WM2664) had similar reductions in the S-phase population with concomitant increases in G1 arrested cells without a sub2N fraction demonstrating that G1T38 was not cytotoxic at the concentrations tested (Supplementary Figure 2). The CDK4/6-independent cell line (A2058) was not affected by treatment with G1T38.

The CDK4/cyclin D1 and CDK6/cyclin D3 complexes are essential for progression from the G1 to the S-phase of the cell cycle through phosphorylation of the tumor suppressor, RB. Once phosphorylated, RB dissociates from cell cycle-promoting transcription factors, allowing them to drive the G1 to S-phase transition [28]. Therefore, CDK4/6 inhibition of RB phosphorylation will arrest cells in G1. To assess the effect of G1T38 on RB phosphorylation, WM2664 cells were treated with varying doses of G1T38 for 24 hours. After treatment, cells were harvested and phosphorylation of RB at the Ser807/811 sites was evaluated via immunoblotting. Phosphorylation of RB at Ser807/811 sites first requires binding of CDK4/6 [3] in order to progress into S-phase [3]. WM2664 cells treated with 30-1000 nM of G1T38 for 24 hours exhibited a complete inhibition of RB phosphorylation compared to vehicle controls (Figure 1D). To determine the kinetics of CDK4/6 inhibition on RB phosphorylation, WM2664 cells were treated with 300 nM G1T38 for 1, 4, 8, 16, and 24 hours. As shown in Figure 1E, treatment with G1T38 reduced RB phosphorylation within 1 hour post-treatment and generated near complete inhibition of RB phosphorylation by 16 hours post-treatment. Taken together, these data demonstrate G1T38 potently and completely inhibits phosphorylation of RB within 24 hours of exposure.

To assess the capacity of G1T38 to inhibit proliferation of tumor cell lines, a broad panel of CDK4/6 dependent and independent lines was evaluated using CellTiter Glo (Table 2). The wide range of tumor lines was chosen to determine if G1T38 could globally inhibit proliferation via CDK4/6. G1T38 produced a robust inhibition of proliferation in a diverse array of tumor cell lines including breast, melanoma, leukemia and lymphoma with EC_50_ concentrations as low as 23 nM. Furthermore, G1T38-associated inhibition in CDK4/6-independent cell lines was greater than 2.5 μM (>2500x IC_50_) demonstrating a functional retinoblastoma pathway is essential for G1T38 activity.

**Table 2:** Growth inhibitory effects of G1T38 on CDK4/6-dependent and CDK4/6-independent cell lines

Cell Line	Tumor Type	RB Status	G1T38 EC_50_ (nM)
MV4-11	B cell leukemia	**+**	23
MCF7	ER+ breast cancer	**+**	52
SupT1	Lymphoma	**+**	57
ZR-75-1	ER+ breast cancer	**+**	61
WM2664	Melanoma	**+**	125
Tom1	Ph1+ leukemia	**+**	232
BV173	Ph1+ leukemia	**+**	296
Daudi	Lymphoma	**+**	784
A2058	Melanoma	**-**	2691
H69	SCLC	**-**	2915
NALM1	Leukemia	**-**	>10000

EC_50_ concentrations (nM) of G1T38 and RB status of tumorigenic cell lines.

### Pharmacokinetics/pharmacodynamics of G1T38 in an ER^+^ breast cancer model

**Figure 2:**
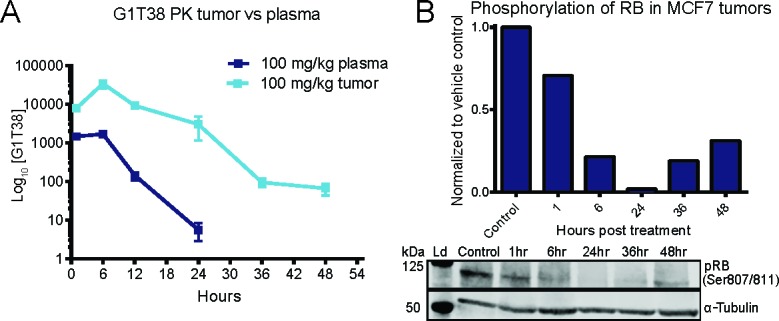
Pharmacokinetics and pharmacodynamics of G1T38 in ER+ breast cancer **(A)** G1T38 concentration in tumor and plasma of C57BL/6 mice after a single oral 100 mg/kg dose of G1T38 (n=3 per time point). **(B)** RB phosphorylation levels in MCF7 tumors from mice treated with a single oral dose of vehicle or 100 mg/kg of G1T38. The western blot shown is a representation of one set of tumors from analyzed samples.

To evaluate the murine PK/PD relationship of G1T38, an ER^+^ MCF7 xenograft model was utilized. Compound concentrations were measured in plasma and tumors from tumor-bearing mice given a single oral dose of G1T38 (100 mg/kg). Based on exposure, G1T38 concentrations were 17-fold higher in the tumor compared to plasma (Figure 2A). The tumor drug concentration was >100 fold that of plasma at 24 hours and ~65 ng/ml of drug remained in the tumor at 48 hours while no drug was detectable in the plasma at this time. To assess the effects of G1T38 on pRB, tumors were harvested and pRB was analyzed by immunoblotting. Phosphorylated RB decreased 30% and 98% relative to the control at 1 hour and 24 hours post-treatment, respectively (Figure 2B). The phosphorylation of RB began to rebound by 36 hours post-treatment and was approximately 31% of control levels at 48 hours post-treatment. The time course of pRB inhibition closely paralleled drug concentrations in the tumor but not the plasma as inhibition of pRB was maintained in the tumor hours after G1T38 was cleared from the plasma. These data suggest oral G1T38 has sufficient mouse PK/PD for assessing efficacy in tumor models.

### G1T38 inhibits breast cancer tumor growth *in vivo*

**Figure 3:**
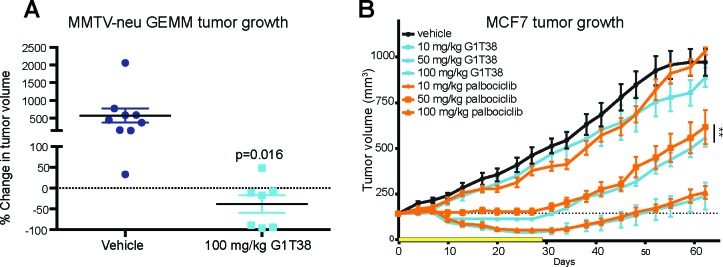
Single agent efficacy of G1T38 in breast cancer **(A)** Mice received 100 mg/kg of G1T38 or vehicle in their diet for 28 days. Food pellets were prepared with the expectation that 100 mg/kg of G1T38 would be orally ingested per day under the parameters that 25 gm mice eat 3.2 gm per day; these parameters were based on previous experience. After 21 days, tumor progression was calculated using the formula: (21 day volume – initial volume)/ initial volume x 100. **(B)** G1T38 or palbociclib efficacy after 28 days of oral treatment (100 mg/kg, 50 mg/kg, 10 mg/kg) in MCF7 xenograft model. Yellow bar represents duration of treatment. Statistics were completed using linear regression analysis of time during treatment (28 days). Error bars represent SEM. **p≤0.01.

We have previously shown that palbociclib is efficacious in the mouse mammary tumor virus (MMTV) - Neu GEMM (Her2/Neu) model, a model that is similar to human HER2-positive (HER2^+^) luminal breast cancer [29]. To assess the antineoplastic effect of G1T38 in this model, mice were given 100 mg/kg of G1T38 in their diet for 28 days with tumor measurements at treatment initiation and every 3 to 4 days during treatment. As shown in Figure 3A, mice treated with G1T38 elicited 38% tumor regression after 21 days of treatment while control animals had a 577% increase in tumor burden over the same treatment period, demonstrating that G1T38 is highly efficacious in this HER2^+^ breast cancer model.

Next, the ER^+^ MCF7 xenograft model was utilized to assess efficacy of G1T38 relative to the first-in-class CDK4/6 inhibitor, palbociclib. Mice were given once daily doses of G1T38, palbociclib, or vehicle by oral gavage at 10, 50, or 100 mg/kg for 28 days. Tumors were measured every 3 to 4 days during treatment for up to 20 days post-treatment. Compared to the vehicle-treated mice, daily treatment with 100 mg/kg of G1T38 or palbociclib showed tumor regression within 10 days in the MCF7 xenograft model (Figure 3B). After 27 days of treatment, tumor growth inhibition (TGI) was observed in the 10, 50, and 100 mg/kg G1T38 cohorts (approximately 12%, 74%, and 90% inhibition, respectively); these data demonstrate that G1T38 can substantially inhibit cell growth in CDK4/6-dependent tumors *in vivo*. Daily oral palbociclib treatment caused an 18%, 66%, and 87% tumor growth inhibition in the 10, 50, and 100 mg/kg dosage cohorts, respectively. Interestingly, at 50 mg/kg, G1T38 was significantly more efficacious than palbociclib. Similar results were seen in the ER^+^ ZR-75-1 breast cancer xenograft model when comparing G1T38 and palbociclib at the 50 mg/kg dose (Supplementary Figure 3).

### G1T38 increases efficacy when combined with multiple targeted therapies in ER^+^ breast cancer

**Figure 4:**
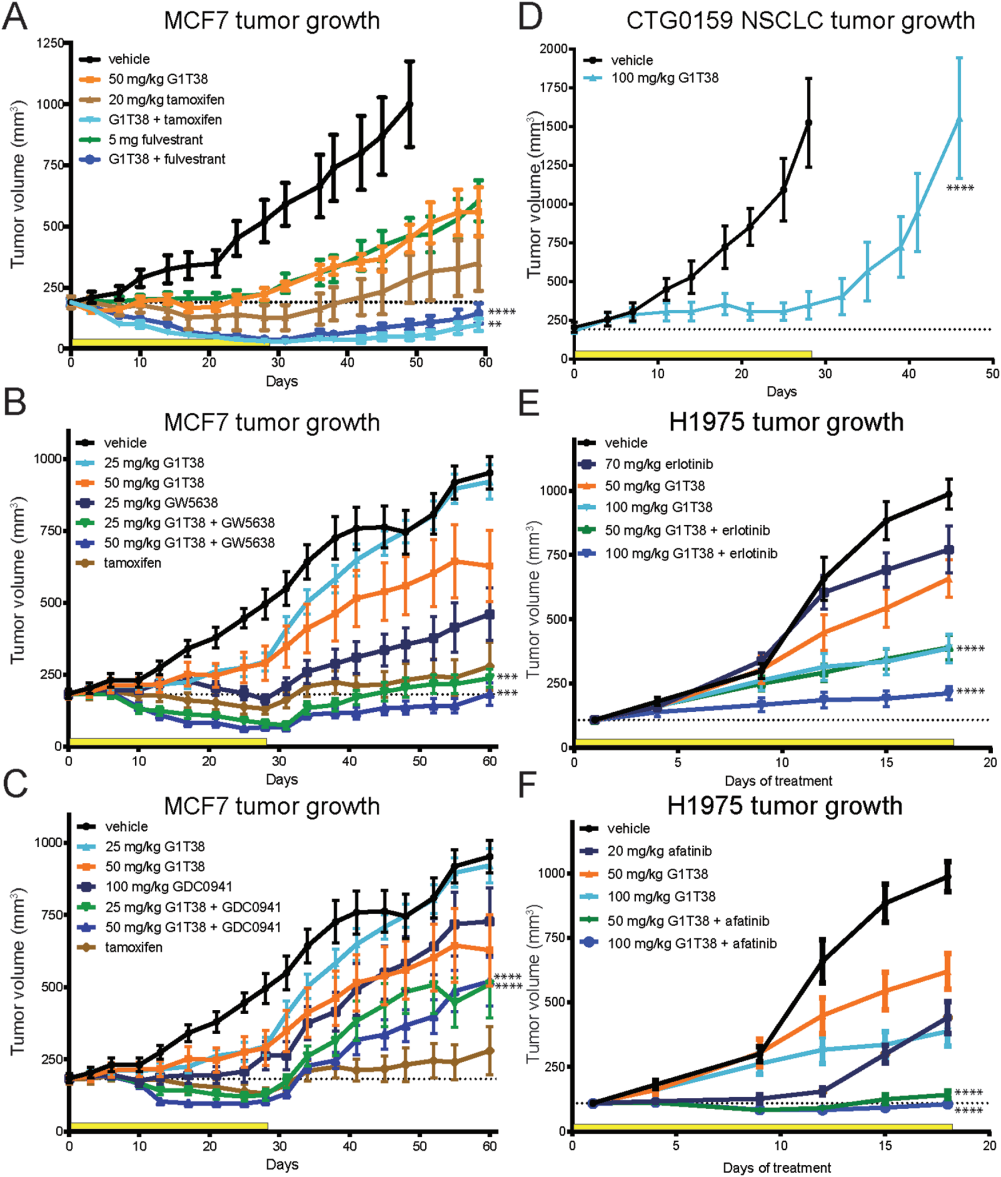
Combination treatments with G1T38 in ER+ breast cancer and NSCLC G1T38 in combination with tamoxifen or fulvestrant **(A)**, SERD (GW5638) **(B)**, PI3K inhibitor (GDC0941) **(C)**, for 28 days of treatment in ER^+^ breast cancer xenograft model (MCF7). **(D)** G1T38 single agent efficacy in NSCLC PDX model CTG-0159 after 28 days of daily 100 mg/kg oral treatment. **(E, F)** G1T38 in combination with oral EGFR inhibitors, erlotinib or afatinib, for 18 days of treatment in EGFR mutant NSCLC xenograft model (H1975). Statistics were completed using linear regression analysis of time during treatment. Error bars represent SEM. Yellow bar represents duration of treatment. **p≤0.01, ***p≤0.001, ****p≤0.0001.

Currently, palbociclib is the only approved CDK4/6 inhibitor and is indicated for ER^+^ breast cancer in combination with letrozole or fulvestrant. To evaluate the antitumor efficacy of G1T38 in combination with endocrine therapy, the ER^+^ breast MCF7 xenograft model was utilized. Mice were treated with 50 mg/kg G1T38, 20 mg/kg tamoxifen, or 5 mg fulvestrant, alone, or combinations of G1T38 + tamoxifen or G1T38 + fulvestrant. After 28 days of treatment, G1T38 + tamoxifen or G1T38 + fulvestrant were significantly more effective than any single agent (G1T38, tamoxifen, or fulvestrant) alone (Figure 4A). Furthermore, the G1T38 combination treatment with either agent resulted in a near complete, sustained tumor regression.

As the only approved selective estrogen receptor degrader (SERD), fulvestrant is administered monthly through intramuscular (IM) injection due to its poor oral bioavailability. For this reason, combination therapy with an orally bioavailable SERD is highly desirable. We assessed the combination of G1T38 with GW5638, a non-commercialized orally available SERD, in the MCF7 model. Here, mice were treated with G1T38 at 25 or 50 mg/kg alone, GW5638 at 25 mg/kg alone or in combination with G1T38 (Figure 4B). As single agents, there was modest TGI in this model, however, the combination of G1T38 and GW5638 led to sustained tumor regression confirming that G1T38 in combination with an orally available SERD increased efficacy in ER^+^ breast cancer potentially obviating the need for IM-administered fulvestrant.

### G1T38 increases efficacy of PI3K inhibitors in ER^+^ breast cancer xenograft model

Targeting the PI3K/AKT axis has been shown to provide an anti-proliferative effect through the reduction of AKT phosphorylation in ER^+^ breast cancer [30]. Preclinical studies have shown that targeting both the CDK4/6 and the PI3K signaling pathways results in tumor regression and reversal of PI3K inhibitor resistance in a MCF7 xenograft model [31]. To assess the effect of combining G1T38 with a PI3K inhibitor, we utilized the MCF7 xenograft model. A commercially available inhibitor of PI3kα/δ, GDC0941, was used for this study. Here, mice were treated with G1T38 at 25 or 50 mg/kg, alone, GDC0941 at 100 mg/kg alone, or in combination. Results show there is modest TGI as single agents. However, the combination of G1T38 and GDC0941 leads to greater efficacy than any of the treatments alone (Figure 4C). These data support combination therapy with G1T38 and a PI3K inhibitor in ER^+^ breast cancer.

### G1T38 is efficacious as a single agent and also increases efficacy in combination with epidermal growth factor receptor (EGFR) inhibitors in EGFR mutated non-small cell lung cancer (NSCLC)

Preclinical studies have shown CDK4/6 inhibition is highly effective at inhibiting the growth of KRAS mutant NSCLC tumors [32]. To evaluate the efficacy of G1T38 in NSCLC, PDX tumor-bearing mice (CTG-0159: stage III primary squamous cell carcinoma, EGFR^WT^) were treated once daily with oral G1T38 at 100 mg/kg for 28 days. Tumors were measured every 3-4 days up to 30 days post treatment. After 28 days of treatment, G1T38 treated mice exhibited 77% TGI with an overall 60% tumor growth delay demonstrating G1T38 alone is highly efficacious in this NSCLC tumor model (Figure 4D).

To evaluate the efficacy of G1T38 in combination with EGFR inhibitors, the H1975 xenograft model was utilized. H1975 is a human NSCLC that harbors the EGFR^L585R/T709M^ mutations rendering resistance to erlotinib treatment [33]. As expected, there was no significant difference between vehicle or erlotinib (70 mg/kg daily) treated mice. G1T38 treatment as a single agent caused dose dependent TGI after 50 and 100 mg/kg daily oral dosing (33.3% and 60.7%, respectively) by 18 days of treatment. Unexpectedly, when 100 mg/kg G1T38 was combined with 70 mg/kg erlotinib for 18 days, there was 80% TGI when compared to vehicle treated cohorts (Figure 4E) demonstrating that combination therapy has greater efficacy than single agent therapy. Interestingly, when G1T38, at any dose, was combined with a 70 mg/kg dose of erlotinib, the resistance to the erlotinib was reversed suggesting the addition of GIT38 reverses the EGFR inhibitor resistance.

Afatinib is an irreversible second generation pan-ErbB inhibitor developed as a first-line treatment of patients with metastatic NSCLC whose tumors harbor EGFR mutations or deletions as well as in patients with metastatic disease that progressed after platinum-based chemotherapy [34]. In the H1975 xenograft model, tumors treated in combination with G1T38 (50 or 100 mg/ kg) and afatinib (20 mg/ kg) did not develop resistance during the 18 days of treatment (Figure 4F). However, within 14 days of treatment, the afatinib, only, cohort began to regrow suggesting the addition of G1T38 to an afatinib regimen can increase the time to resistance of the EGFR inhibitor in NSCLC.

### Rapid clearance of G1T38 from plasma versus palbociclib leads to reduced myelosuppression in murine bone marrow

**Figure 5:**
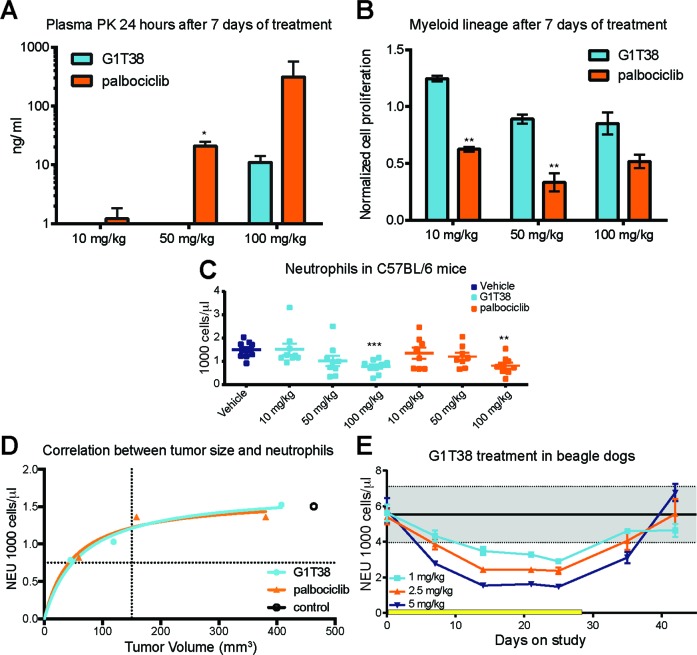
Comparison of myeloid precursor proliferation following G1T38 and palbociclib treatment **(A)** Plasma concentrations of G1T38 or palbociclib 24 hours post 7 days treatment. **(B)** 12 hours post 7 days of treatment, bone marrow was harvested and proliferation (EdU incorporation) was measured in myeloid progenitors (Mac1^+^ Gr1^+^). **(C)** G1T38 and palbociclib neutrophil counts after 28 days of treatment in C57BL/6 mice. **(D)** Neutrophil counts and tumor volume in mice after 28 days of palbociclib or G1T38 daily oral treatment as previously described was analyzed using Michaelis-Menten nonlinear regression analysis. Vertical dotted line indicates size of tumor at treatment initiation. Horizontal dotted line indicates level of severe neutropenia in animals (50% of control neutrophil counts). Open circle indicates level of neutrophils and size of tumor after 28 days of daily oral vehicle treatment. **(E)** Neutrophil counts in beagle dogs during and after 28 days of G1T38 daily oral treatment (n=10; 5 males, 5 females). Yellow bar represents duration of treatment. Error bars represent SEM. *p≤0.05, **p≤0.01, ***p≤0.001.

To better understand the relationship of CDK4/6 inhibition and neutropenia, C57BL/6 mice were treated for 7 days with G1T38 or palbociclib at 10, 50, or 100 mg/kg. After 7 days of daily oral dosing, palbociclib concentrations in plasma 24 hours after the final dose were ~300 ng/ml (approximately 600 nM; Figure 5A). This is significantly higher than the concentration necessary to inhibit cellular proliferation in most RB-dependent cell lines tested (Table 2). In contrast, G1T38 plasma concentrations at 24 hours after the last dose were 11 ng/ml (approximately 22 nM); well below the concentration necessary to maintain a G1 arrest. This suggests that palbociclib-induced neutropenia may be due to accumulation of the drug resulting in persistent inhibition of CDK4/6 in the bone marrow, thus preventing the recovery of bone marrow proliferation prior to subsequent doses.

To evaluate whether higher palbociclib exposures account for a more sustained inhibitory effect on the hematopoietic system than G1T38, mice were treated with various doses of G1T38 or palbociclib for 7 days and proliferation of the myeloid progenitor lineage was measured 12 hours post-treatment via 5-ethynyl-2′-deoxyuridine (EdU) incorporation, a time point where previous experiments demonstrate bone marrow proliferation nadirs after a single dose of either compound. G1T38-treated mice showed no differences in myeloid progenitor proliferation in any treatment cohort when compared to vehicle, while palbociclib treatment led to more than a 50% reduction in proliferation in both the 50 and 100 mg/kg cohorts (Figure 5B). These data indicate that between doses, the longer exposure of palbociclib resulted in drug concentrations that were above the threshold necessary to maintain G1 arrest of bone marrow progenitor cells resulting in sustained inhibition of myeloid progenitors. In contrast, due to the minimal G1T38 compound in the plasma at 24 hours, the bone marrow seem to have more time to recover from CDK4/6 inhibition between doses suggesting that continuous daily dosing may be achievable in cancer patients.

To compare the effects of G1T38 and palbociclib on neutrophils during daily treatment, neutrophils were measured at Days 0, 14, and 28. As shown in Figure 5C, a dose-dependent decrease in neutrophils was observed with maximal effect in the 100 mg/kg G1T38 cohort with a 48% decrease in neutrophils demonstrated after 28 days of once daily oral dosing. The decrease in neutrophils was similar in the palbociclib-treated cohorts (Figure 5C). Thus, it appears the difference between palbociclib and G1T38 inhibition of myeloid progenitors in murine bone marrow is not sufficient to further reduce the number of circulating neutrophils. Therefore, in mice, the therapeutic index (TI) (the difference between tumor efficacy and neutropenia) for dosing G1T38 and palbociclib appears similar. To confirm, an E_max_ model was generated to compare neutrophil counts to tumor efficacy in both G1T38 and palbociclib treated cohorts. As shown in Figure 5D, there is a close correlation between G1T38 and palbociclib suggesting that both drugs equally affect tumor proliferation and neutrophil counts, confirming there is no difference in the TI in mice.

However, in GLP 28-day toxicology studies in beagle dogs, neutrophils decreased rapidly in a dose dependent manner during the first 14 days of G1T38 treatment. However, from 14 to 25 days, cells reached a steady state level, which was readily reversible once dosing was stopped (Figure 5E). While the decrease in neutrophil count was higher as the dose increased, the neutrophil count at each dose level did not decrease further once the nadir level was achieved at 14 days. These data suggest that continuous daily dosing, without a break, may be achievable in cancer patients.

## DISCUSSION

The therapeutic importance of CDK4/6 as a target has been established by palbociclib (IBRANCE®), a first-in-class CDK4/6 inhibitor, which is approved for the treatment of hormone receptor (HR)-positive and human epidermal receptor 2 (HER2)-negative breast cancer in combination with the aromatase inhibitor, letrozole, or the SERD, fulvestrant [35]. Although palbociclib has proven efficacious, daily treatment causes severe neutropenia, which necessitates a 7-day treatment holiday [23, 24]. Palbociclib-induced neutropenia may lead to an increased risk of infections and the treatment holiday may lead to tumor outgrowth and emergence of drug resistance. Therefore, a next generation CDK4/6 inhibitor should inhibit CDK4/6-dependent tumor growth while minimizing neutropenia, thereby reducing the need for treatment holidays and decreasing the risk of inducing drug resistance.

Here, we have identified and characterized G1T38; a potent and highly selective CDK4/6 inhibitor with antiproliferative activity in CDK4/6- dependent cells and tumors. Biochemical profiling against the CDK family showed G1T38 was most potent on CDK4/ cyclin D1 (1 nM), CDK6/ cyclin D3 (2 nM) and CDK9/ cyclin T (28 nM). G1T38 was >1000 fold selective for CDK4/ cyclin D1 than CDK2/cyclin E and CDK2/cyclin A. When profiled against a broader panel of kinases, G1T38 was highly selective against the majority of kinases. We investigated whether G1T38 might be affecting pathways regulated through CDK9 inhibition, but did not see an effect on RNA polymerase II. Interestingly, G1T38 is highly potent against FLT3 (D835V) and FLT3 (ITD, D835V) while not against FLT3 wild type protein. While high levels of wild-type expression is observed in acute myelogenous leukemia and B-cell acute lymphoblastic leukemia, the mutant forms are manifested as leukemic driver mutations resulting in uncontrolled receptor activation and are associated with negative outcome [36]. We did not observe a FLT3 specific effect on myelogenesis with G1T38 outside the CDK4/6 inhibitory effect on bone marrow. In WM2664 cells, G1T38 produced a dose dependent G1 arrest with an EC_50_= 20 nM. This G1 arrest was maintained through at least 300 nM. The G1 arrest was concomitant with inhibition of pRB. However, in cells where the RB pathway was disrupted, G1T38 had no cell cycle effect through 2.5 μM demonstrating that the G1 arrest was specific for CDK4/6-dependent cells. Additionally, G1T38 was not toxic to the cells, as we observed no decrease in cell proliferation, morphological change or increase in sub 2N in cell populations where the RB pathway was disrupted.

G1T38 has a relatively short plasma half-life with minimal accumulation upon repeat dosing. In tumor bearing mice, we found that while G1T38 was readily cleared from the plasma, it appeared to accumulate in tumors. We found that the concentrations of G1T38 in tumors closely correlated with inhibition of tumor pRB, demonstrating that G1T38 in the tumor could inhibit the CDK4/6/RB pathway and sustain tumor cell growth inhibition after G1T38 plasma concentrations were <1 ng/ml. Assuming the bone marrow compartment has similar drug concentrations to circulating drug in the plasma, the differential drug concentrations in the bone marrow and tumor could allow for a larger TI. These unique PK/PD properties may allow for continuous daily dosing in the clinic

Previously, we reported palbociclib significantly inhibited HER2^+^ tumor growth in the MMTV-HER2-neu GEMM when orally dosed for 28 days [29]. Here, we found that G1T38 was highly efficacious in this model demonstrating that G1T38 and palbociclib are equally effective as single agents and confirming that HER2^+^ tumors are sensitive to CDK4/6 inhibition. Studies comparing G1T38 to palbociclib in a human ER^+^ mouse xenograft model demonstrate equivalent or better single agent efficacy (Figure 3B and Supplementary Figure 3). When comparing the plasma exposures of G1T38 and palbociclib to efficacy (% TGI) in a nonlinear regression model, G1T38 had a 3.8 fold lower EC_50_ than palbociclib (Supplementary Figure 4). This most likely reflects both the greater biochemical and cellular potency of G1T38 (IC_50_=1 nM vs. IC_50_ =10 nM for palbociclib; G1T38 EC_50_=2-3 fold < palbociclib) and difference in PK properties of the compounds.

Preclinical studies have generally shown that CDK4/6 inhibitors have greater efficacy when given in combination with various growth signaling inhibitors (GSI) [37]. Likewise, in clinical studies, combination therapies targeting CDK4/6 and ER signaling pathways have been shown to significantly increase progression free survival [38, 39]. Thus, further development of CDK4/6 inhibitors will most likely be done in combination with GSIs. In multiple combination studies targeting CDK4/6, ER, and PI3K pathways in human ER^+^ mouse xenograft models, G1T38 was significantly more efficacious when combined with the GSI then as a single agent (Figure 4). In most of these studies, tumors began proliferating within a few days once dosing was stopped. However, the combination of G1T38 with GW5638, fulvestrant or tamoxifen was particularly efficacious and TGI persisted for several weeks once dosing was stopped. Furthermore, the addition of G1T38 increased the efficacy of an anti-estrogen treatment independent of G1T38 dose (Figure 4B). These data suggest lower doses of CDK4/6 inhibitors and GSIs could be used in combination to decrease toxicity while remaining highly efficacious.

In NSCLC, combination studies targeting CDK4/6 and EGFR signaling also showed increased efficacy when compared to single agent therapy. In fact, the combination of G1T38 (50 mg/kg) and erlotinib was equally efficacious as the G1T38 (100 mg/kg) alone cohort. Interestingly, G1T38 also reversed erlotinib-resistance. While it has been previously shown that JAK2 inhibition re-sensitizes NSCLC to EGFR inhibitors and G1T38 inhibits JAK2, the IC50 was >500 nm; a concentration that is too high for any biological relevance [40]. Additionally, upregulation of AXL leads to EGFR inhibitor resistance in NSCLC causing activation of MAPK, AKT and NFkB pathways [41]. CDK4/6 inhibition has been shown to affect signaling in all three pathways [42–44] suggesting a possible mechanism whereby G1T38 reverses the resistance of erlotinib in EGFR mutant NSCLC.

As we have previously shown that bone marrow hematopoietic stem and progenitor cells are CDK4/6 dependent [He S, Roberts PJ, Sorrentino JA, et al., in press]. Thus, the neutropenia observed in the clinic with CDK4/6 inhibitors is a consequence of the on-target effect of prolonged drug exposure, which has resulted in dosing regimens that require at least a 7-day treatment holiday to allow recovery of neutrophils. These studies also indicate a narrow TI between tumor efficacy and neutropenia. This is most likely due to the long half-life and accumulation of palbociclib with repeat dosing. Thus drug exposure may be prolonged above the threshold to maintain arrest of bone marrow myeloid progenitors, resulting in more severe neutropenia. In fact, palbociclib has a half-life of >24 hours in humans with a median accumulation ratio of 2.4 (range 1.5-4.2) [35]. To better address the narrow TI, G1T38 was designed to have minimal accumulation on repeat dosing while maintaining tumor efficacy. In mice, the effects of G1T38 and palbociclib on neutrophils were similar and reflected the nearly identical elimination half-lives of these compounds. However, unlike G1T38, sustained plasma concentrations of palbociclib at 24 hours after administration caused a decrease in myeloid progenitors, i.e., a more sensitive target population than circulating neutrophils. The minor difference observed in mouse PK and myeloid progenitors appears to be exacerbated in larger mammals leading to longer half-lives, accumulation of drug, and increased sensitivity to CDK4/6 inhibition (Supplementary Table 2). The toxicity of palbociclib was only evaluated for 21 days in repeat dose GLP toxicology studies in rat and dog, rather than the standard 28-day studies [45], whereas, G1T38 was assessed in 28-day repeat-dose GLP toxicity studies in rat and dog. With oral administration of G1T38, anticipated reduction of bone marrow cellularity and lymphocyte depletion was observed leading to dose-related decreases in hematopoiesis. However, at clinically relevant doses, these effects were not sufficient to limit dosing to less than 28 days. There were no reported increases in the incidence of infection or bleeding throughout dosing and inhibition of hematopoiesis was found to be reversible upon discontinuing dosing of G1T38 during the recovery period. Additionally, there was no accumulation of G1T38 as measured by PK on repeat dosing through 28 days in either rats or dogs (Supplementary Table 3). These data suggest G1T38 has the potential to be dosed continuously in humans at an efficacious level.

In summary, we have identified and characterized G1T38 as a novel, potent, selective and orally bioavailable small molecule inhibitor of CDK4/6 that shows significant efficacy in CDK4/6 dependent tumors by effectively inhibiting tumor growth without causing severe neutropenia. G1T38 may, therefore, be an optimal therapeutic for daily use as an oral antineoplastic agent, allowing for a greater TI than palbociclib. G1T38 has recently completed testing in human subjects in a Phase 1 healthy volunteer study to assess safety, PK, and tolerability in 75 healthy subjects [46]. We are currently evaluating G1T38 (in combination with Faslodex) in a Phase 1b/2a trial in ER+, HER- breast cancer patients [47].

## MATERIALS AND METHODS

### Chemicals

G1T38: 2′-((5-(4-isopropylpiperazin-1-yl)pyridin-2-yl)amino)-7′,8′dihydro-6′H-spiro[cyclohexane1,9′pyrazino[1′,2′:1,5]pyrrolo[2,3-d]pyrimidin]-6′-one di-hydrochloride was synthesized and characterized for purity and identity as an HCl salt at ChemoGenics BioPharma, LLC under the direction of G1 Therapeutics, Inc. GW5638: was synthesized at PharmAdvance (Jiangyin, Jiangsu, P.R. China).

### Nanosyn CDK biochemical *in vitro* assay

Compounds were tested in CDK1/CYCLIN B1, CDK2/CYCLIN A, CDK2/CYCLIN E, CDK4/CYCLIN D1, CDK6/CYCLIN D3, CDK5/p25, CDK5/p35, CDK7/CYCLIN H-MAT1, and CDK9/CYCLIN T kinase assays (Nanosyn, Inc.; Santa Clara, CA). The assays were completed using microfluidic kinase detection technology (Caliper Assay Platform). The compounds were tested in 12-point dose response format in singlicate at the Km for ATP. Phosphoacceptor substrate peptide concentration used was 1 μM and Staurosporine was used as the reference compound for all assays.

### KINOME*scan* primary screen and K_d_ determination

G1T38 was profiled at DiscoveRx (Fremont, CA) using their KINOMEscan and scanMAX screening technology [48]. Briefly, G1T38 was tested at 100 and 1000 times the biochemical IC_50_ as described in Figure 1B. All target kinases that responded to greater than 90% inhibition were tested as individuals for K_d_ determination as described in Supplementary Table 1.

### Cell lines

Cell lines were obtained from American Type Culture Collection (ATCC; Manassas, VA) or Leibniz-Institut DSMZ-Deutsche Sammlung von Mikroorganismen und Zellkulturen GmbH (Braunscheig, Germany). HS68 (CRL-1635™) and A2058 (CRL-11147™) cells were grown in Dulbecco's Modified Eagle's Medium (DMEM) (Life Technologies/ Thermo Fisher Scientific, (Waltham, MA) containing 10% fetal bovine serum (HyClone/ GE Healthcare; Pittsburgh, PA) and 1x Glutamax (Life Technologies). MCF-7 (HTB-22™) and WM2664 (CRL-1676™) cells were grown in Eagle's Modified Dulbecco's Medium (EMEM) (Life Technologies) containing 10% fetal bovine serum and 1x Glutamax. ZR-75-1 (CRL-1500™), NCI-H69 (HTB-119™), Daudi (CCL-213™) and SUP-T1 (CRL-1942™) were grown in RPMI-1640 (CELLGRO/ Corning; Corning, NY) containing 10% fetal bovine serum and 1x Glutamax. NALM-1 (CRL-1567™) cells were grown in RPMI-1640 (CELLGRO) containing 15% fetal bovine serum and 1x Glutamax. MV-4-11 (CRL-9591) cells were grown in Iscove's Modified Dulbecco's Medium (IMDM) (ATCC). BV173 (ACC-20) and Tom-1 (ACC-578) cells were grown in RPMI-1640 (CELLGRO) containing 20% heat inactivated fetal bovine serum (Hyclone) and 1 x Glutamax. Heat inactivation of fetal bovine serum was performed by warming serum to 37°C with mixing, then placing the serum in 56°C water bath for 30 minutes with mixing every 15 minutes, followed by cooling immediately in ice bath. Serum was stored at -20°C until ready for use. Cell lines were authenticated by ATCC in September 2105 and Genetica DNA Laboratories (Burlington, NC) in October 2016 using short tandem repeat (STR) profiling.

### Western blot analysis for pRb and total Rb

WM2664 cells were either treated for dose response (3, 10, 30, 100, 300 or 1000 nM) for 24 hours or a time course (1, 4, 8, 16 or 24 hours) with 300 nM G1T38. Whole cell extracts were prepared using 1x radioimmunoprecipitation assay buffer (RIPA) (ThermoFisher) containing 1x HALT^®^ protease and phosphatase inhibitors (ThermoFisher). Total protein concentration was determined by using the bicinchoninic acid (BCA) Protein Assay Kit (PIERCE/ ThermoFisher), according to manufacturer's instructions. 20 micrograms of protein were heat denatured for 10 minutes at 70°C and resolved by Novex^®^ NuPAGE® SDS-PAGE gel system (ThermoFisher) at 200 volts, constant current and transferred to 0.45 μm nitrocellulose membrane (Life Technologies) in 1 x Transfer buffer (Life Technologies) plus 20% methanol (Sigma-Aldrich (St. Louis, MO) by electroblotting at 35 volts, constant current. Membranes were blocked in LiCor Membrane Blocking Buffer (Lincoln, NE) and incubated overnight with either rabbit anti-pRb (Ser807/811, CST-9308) antibody (Cell Signaling Technology (Danvers, MA) at a 1:500 dilution or mouse anti-Rb (CST-9309) at a 1:2,000 dilution and mouse α-tubulin (CST-3873) antibody (Cell Signaling Technology) at a 1:1,000 dilution, as a loading control. Secondary antibodies (LiCor) were goat anti- rabbit (680RD) and goat anti-mouse (800CW) at a 1:15,000 dilution. Blots were incubated for one hour, washed and imaged using LiCor ImageStudio software (Version 4.0.21).

### Cell proliferation

SupT1, Daudi, MCF7, ZR-75-1, A2058, WM2664, and H69 cells were seeded at 1000 cells per well; MV-4-11 and BV173 cells were plated at 4000 cells per well; Tom-1 cells were plated at 8,000 cells per well; NALM-1 cells were plated at 20,000 cells per well in Costar 3903 96 well plates. After 24 hours, plates were dosed with G1T38 at a nine-point dose concentration from 10 μM to 1 nM. Cell viability was determined after four or six days using the CellTiter-Glo® assay (Promega; Madison, WI) following manufacturer's recommendations. Plates were processed on BioTek (Winooski, VT) Synergy2 multi-mode plate reader and data analyzed using GraphPad (San Diego, CA) Prism 5 statistical software.

### Ethics statement

*In vivo* investigation has been conducted in accordance with the ethical standards and according to the Declaration of Helsinki and according to the international guidelines and has been approved by each contract research organization's institutional review board.

### MCF-7 breast cancer xenograft model

South Texas Accelerated Research Therapeutics (START; San Antonio, Texas) evaluated the antitumor activity of G1T38 in a START Cell-Based Xenograft (START-CBX) tumor model, MCF-7, representing human breast cancer. Data collected from this efficacy study included animal weights, observations, and tumor dimensions. This information was used to determine agent tolerability based on weight change and gross physiologic changes, and anticancer activity based on tumor growth inhibition or regression. Study endpoint was Day 60. MCF-7 cells were harvested from culture and injected into immune-deficient mice and the study initiated at a mean tumor volume of approximately 150-250 mm^3^. In all studies, G1T38 and palbociclib was given as a daily oral treatment at 10, 25, 50 or 100 mg/kg. Tamoxifen was administered subcutaneous (s.c.) at 20 mg/kg M-F, fulvestrant was administered s.c. 5 mg per mouse once a week, GW5638 was administered orally at 25 mg/kg daily and GDC0941 (Selleckchem) was administered orally at 100 mg/kg daily for the duration of each study. Tumor growth inhibition (%TGI) was calculated using the following equation: %TGI=(1-(((TV_tmtfinal_-TV_tmtinitial_))/((AVERAGE(TV_ctlfinal_))-(AVERAGE(TV_ctlinital_)))))*100. All protocols were IACUC approved and experiments were completed at START.

### Her2Neu breast cancer GEMM

Female MMTV-NEU mice (Jackson Labs Strain 002376) were used to test the efficacy of G1T38 (100 mpk, medicated diet). Drug was incorporated into diet by Research Diets (New Brunswick, NJ) using the following parameters; 25 gm mouse eating 3.2 gm of food/day. Mice were monitored for tumor development by palpating them weekly as per University of North Carolina at Chapel Hill Lineberger Mouse Phase 1 Unit (MP1U) protocol. At time of treatment, body composition was assessed and weight measurements (in grams) were recorded and used as a measure of gross toxicity. Mice were monitored until euthanized at predetermined humane endpoints per approved University of North Carolina at Chapel Hill IACUC protocols.

### CTG0159 PDX NSCLC model

Female nude mice were implanted with NSCLC PDX CTG0159 tumor. Mice were then randomized into treatment groups and dosing initiated once tumors reached a volume that fell within the range of 150-300 mm^3^. 100 mg/kg G1T38 or vehicle was orally administered for 28 consecutive days. Tumors were measured twice weekly until mice reached tumor burden of 1500 mm^3^. All protocols were IACUC approved and experiments were completed at Champions Oncology (Baltimore, MD).

### H1975 NSCLC xenograft model

Female NCI Ath/nu mice were implanted with H1975 NSC lung adenocarcinoma model. Once tumors reached an average size of 100- 150 mm^3^, mice were randomized into treatment cohorts. Mice were orally administered daily afatinib (20 mg/kg, Genentech/OSI), erlotinib (70 mg/kg, LC Labs), or G1T38 (50 or 100 mg/kg), as single agents or in combination (G1T38 + erlotinib or G1T38 + afatinib) for the duration of the study. Tumors were measured twice weekly until mice reached tumor burden of 1500 mm^3^. All protocols were IACUC approved and experiments were completed at Charles River Laboratories (CRL; Research Triangle Park, NC).

### *In vivo* G1T38 murine pharmacokinetic (PK) assay

For PK analysis comparing G1T38 levels in tumor and plasma, MCF7 tumor bearing mice were given a single oral gavage at 100 mg/kg of G1T38 and blood via cardiac puncture and tumors were harvested at 0 (predose), 6, 12, 24, 36, 48 hours post treatment. For 7-day continuous oral PK analysis, C57BL/6 mice were administered daily oral gavages of 10, 50 or 100 mg/kg of G1T38 or palbociclib for 7 consecutive days. Blood was collected from treated mice via cardiac puncture 24 hours post drug administration (3 mice/dose/time-point). Plasma G1T38 concentration was determined by LC-MS/MS following standard protocols. PK analyses on plasma G1T38 concentration were performed in Watson (v7.3.0.01, Thermo Inc., Philadelphia, PA).

### Inhibition of Rb phosphorylation in MCF-7 tumors

Mice carrying MCF-7 xenograft tumors were orally dosed once with vehicle or G1T38 at 100 mg/kg. Tumors were harvested at 1, 6, 24, 36, or 48 hours, rinsed in phosphate buffered saline -calcium magnesium free (PBS-CMF) and snap frozen in liquid nitrogen. Tumors were then lysed in RIPA buffer containing protease and phosphatase inhibitors on ice and homogenized using Biomasher II mini-pestle (Kimble Chase Life Sciences, Vineland, NJ) until fully dissociated. Protein concentration determination and western blot analysis methods for pRB Ser 807/811 as previously described.

### Peripheral blood analysis of G1T38 and palbociclib in mice

C57BL/6 female mice were given daily oral doses of vehicle, palbociclib or G1T38 at 10, 50 or 100 mg/kg for 28 days. CBCs were measured every two days starting on day six. Data reported are from day 6 (Platelets), day 10 (white blood cells [WBC], neutrophils [Neu], lymphocytes[Lymph]), or day 16 (red blood cells [RBC]). All protocols were IACUC approved and experiments were completed at Integrated Laboratory Systems (Research Triangle Park, NC).

### CBC and PK analysis in G1T38 treated beagle dogs

Beagle dogs (5/ sex) were dosed once daily for 28 consecutive days via oral gavage. The dose levels were 1, 2.5 and 5 mg/kg/day and administered at a dose volume of 5 mL/kg. Hematology samples (1ml/ time point) were collected in K_3_EDTA tubes twice pre-test and then on days 7, 14, 21, and 25 during the dosing period and on days 35, and 42 during the recovery period. Complete blood counts were performed and results reported. All studies were conducted utilizing methods and protocols in accordance with IACUC approval at MPI Research (Mattawan, MI).

### EdU incorporation in G1T38 and palbociclib treated mice

C57BL/6 mice were administered daily oral gavages of 10, 50 or 100 mg/kg of G1T38 or palbociclib for 7 consecutive days. Eleven hours later, each mouse was given a single IP injection of 10 mg/kg EdU and femurs harvested 1 hours later (3 mice/dose/time-point). All protocols were IACUC approved and experiments were completed at Integrated Laboratory Systems. Bone marrow cells were isolated by flushing the femurs using ice-cold staining medium (1× Ca^2+^- and Mg^2+^-free Hank's balanced salt solution (HBSS, Gibco) supplemented with 10 mM EDTA (Corning) and 2% heat-inactivated bovine serum (Gibco)) and filtered through 40-μm nylon mesh (ThermoFisher). The cells were then fixed in 5% formalin for 10 minutes on ice and permeabilized in staining medium plus 0.1% saponin for 10 minutes at room temperature. EdU staining was completed using manufactures’ instructions (Life Technologies). Flow cytometry analysis of stained cells was completed on a LSR II 3-laser flow cytometer (Becton-Dickinson), and recorded data was analyzed in FlowJo 10.0.8 software.

## SUPPLEMENTARY MATERIALS FIGURES AND TABLES


