# Supplementary material for: Preclinical development of G1T38: A novel, potent and selective inhibitor of cyclin dependent kinases 4/6 for use as an oral antineoplastic in patients with CDK4/6 sensitive tumors
Source: Oncotarget. 2017 Mar 15;8(26):42343–58. doi: 10.18632/oncotarget.16216 (PMC5522071; doi:10.18632/oncotarget.16216)
Supplement: Supplementary file 1 [file oncotarget-08-42343-s001.pdf]

Preclinical development of G1T38: A novel, potent and selective inhibitor of cyclin dependent kinases 4/6 for use as an oral antineoplastic in patients with CDK4/6 sensitive tumors

SUPPLEMENTARY DATA

MATERIALS AND METHODS

Western blot analysis for Rpb1 CTD

HS68 cells were treated for dose response (3, 10, 30, 100, 300 or 1000 nM) for 24 hours. Western blot method performed as described previously. Upon blocking, membranes were incubated overnight with rabbit anti-pRpb1 CTD (SER2, CST-13499) or mouse anti-Rpb1 CTD (CST-2629) antibody (Cell Signaling Technology (Danvers, MA) at a 1:1000 dilution and mouse α-tubulin (CST-3873) antibody (Cell Signaling Technology) at a 1:1,000 dilution, as a loading control. Secondary antibodies (LiCor) were Goat anti- rabbit (680RD) and Goat anti-mouse (800CW) at a 1:15,000 dilution. Blots were incubated for one hour, washed and imaged using LiCor ImageStudio software (Version 4.0.21).

Cell cycle analysis

HS68, WM2664, and A2058 cells were treated for 24 hours with G1T38 at 10, 30, 100, 300, or 1000 nM final concentration. Cells were harvested and fixed in ice-cold methanol (Sigma) and stored at -20°C. Fixed cells were stained with 20 µg propidium iodide (Sigma), 50 µg RNase A (Sigma) in PBS-CMF + 1% Bovine Serum Albumin (BSA), Fraction V (Fisher Scientific). Samples were processed on Cyan ADP Analyzer (Beckman Coulter (Indianapolis, IN), and cell cycle analysis was completed using FlowJo software (Version 10.0.8; Tree Star (Ashland, OR).

ZR-75-1 breast cancer xenograft model

Woodland Pharmaceuticals (Shrewsbury, MA) evaluated the antitumor activity of G1T38 in a Cell-Based Xenograft (CBX) tumor model, ZR-75-1, representing human breast cancer. Data collected from this efficacy study included animal weights, observations, and tumor dimensions. This information was used to determine agent tolerability based on weight change and gross physiologic changes, and anticancer activity based on tumor growth inhibition or regression. Study endpoint was Day 60. ZR-75-1 cells were harvested from culture and injected into immune-deficient mice and the study initiated at a mean tumor volume of approximately 150 mm^3. Each cohort consisted of 10 tumor-bearing mice. In all studies, G1T38 and palbociclib was given as a daily oral treatment at 10, 25, 50 or 100 mg/kg. All protocols were IACUC approved and experiments were completed at Woodland Pharmaceuticals.

PK analysis in G1T38 treated wistar han rats

Wistar Han rats (16/ sex) were dosed once daily for 28 consecutive days via oral gavage. The dose levels were 2.5, 5.0 and 15 mg/kg/day and administered at a dose volume of 5 mL/kg. PK time points were collected on days 1 and 28 at alternating time points from two cohorts of three animals/ sex at approximately 0.5, 1, 2, 4, 8, 12, 18, 24, and 36 hours (Day 28 only) postdose. All studies were conducted utilizing methods and protocols in accordance with IACUC approval at MPI Research (Mattawan, MI).

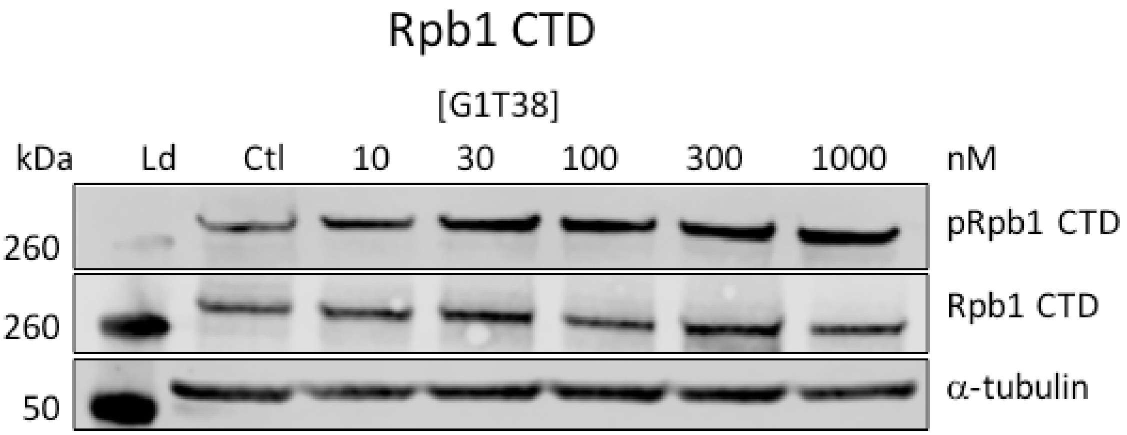

Supplementary Figure 1: HS68 cells treated with G1T38 for 24 hours show no decrease in Rpb1 CTD (SER2) phosphorylation in dose-dependent fashion with no effect on total Rpb1 CTD protein. α-tubulin was used as the loading control.

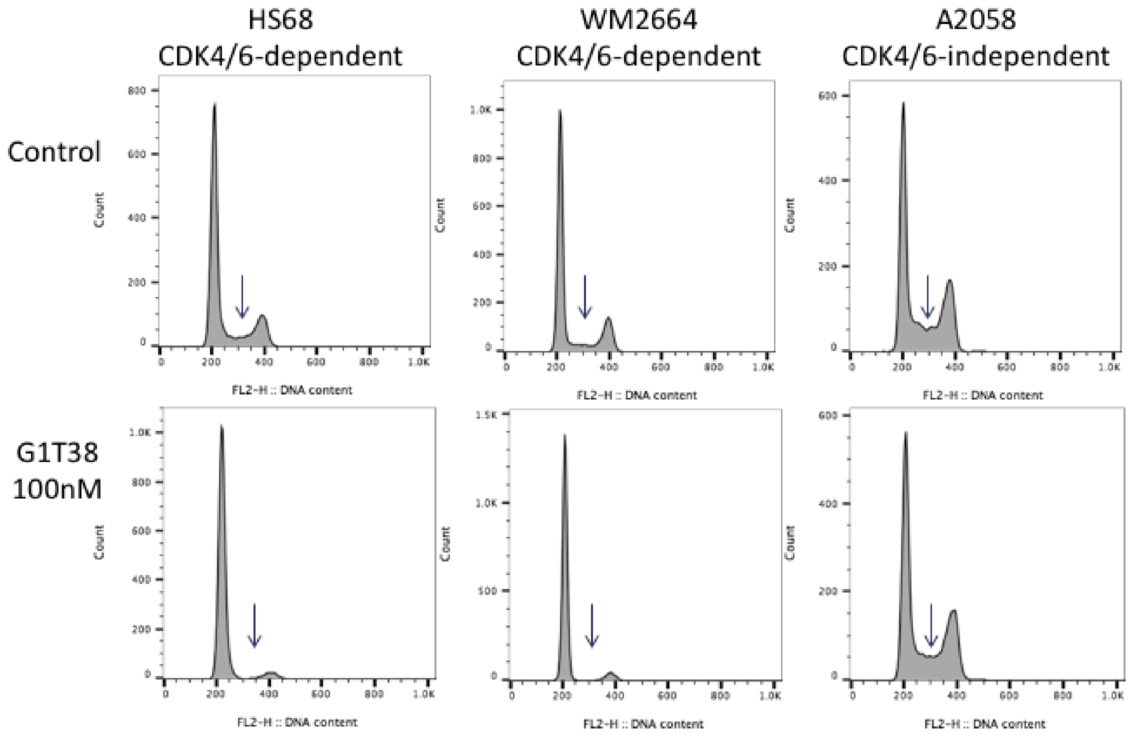

Supplementary Figure 2: 24-hour treatment with G1T38 causes a loss of the S-phase (indicated by arrow) in CDK4/6-dependent cell lines (HS68 and WM2664), but not in the CDK4/6 independent cell line (A2058) as measured by propidium iodide staining.

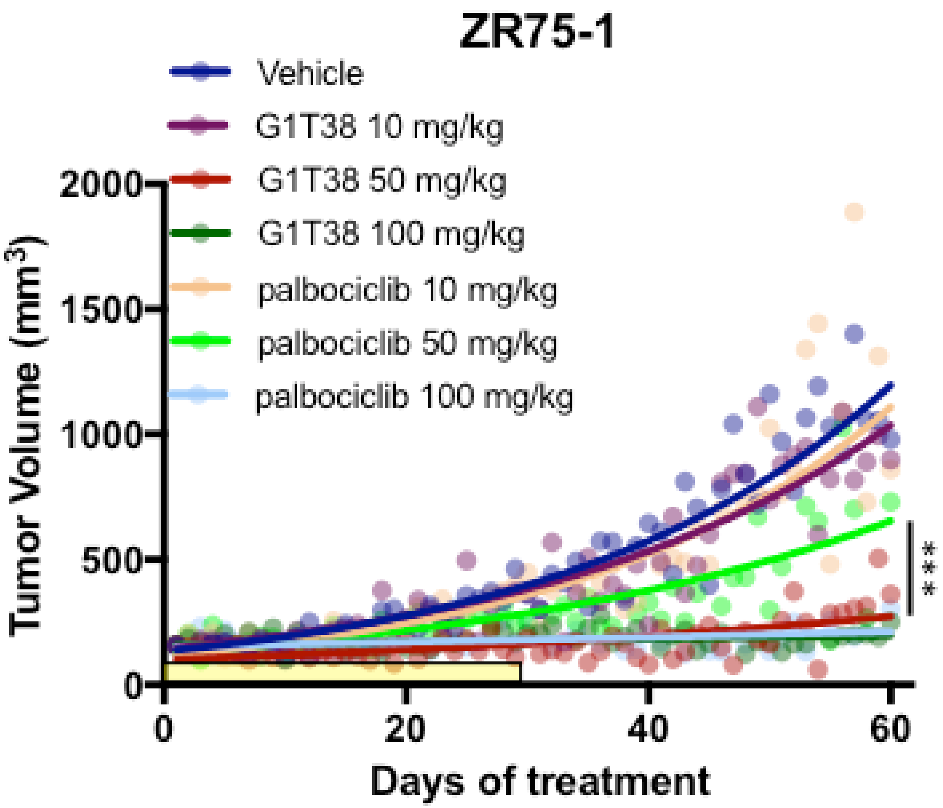

Supplementary Figure 3: G1T38 or palbociclib efficacy after 28 days of oral treatment (100 mg/kg, 50 mg/kg, 10 mg/kg) in ZR-75-1 xenograft model. Yellow bar represents duration of treatment. Statistics were completed using linear regression analysis of time during treatment (28 days) comparing equivalent doses. ***p≤0.0001.

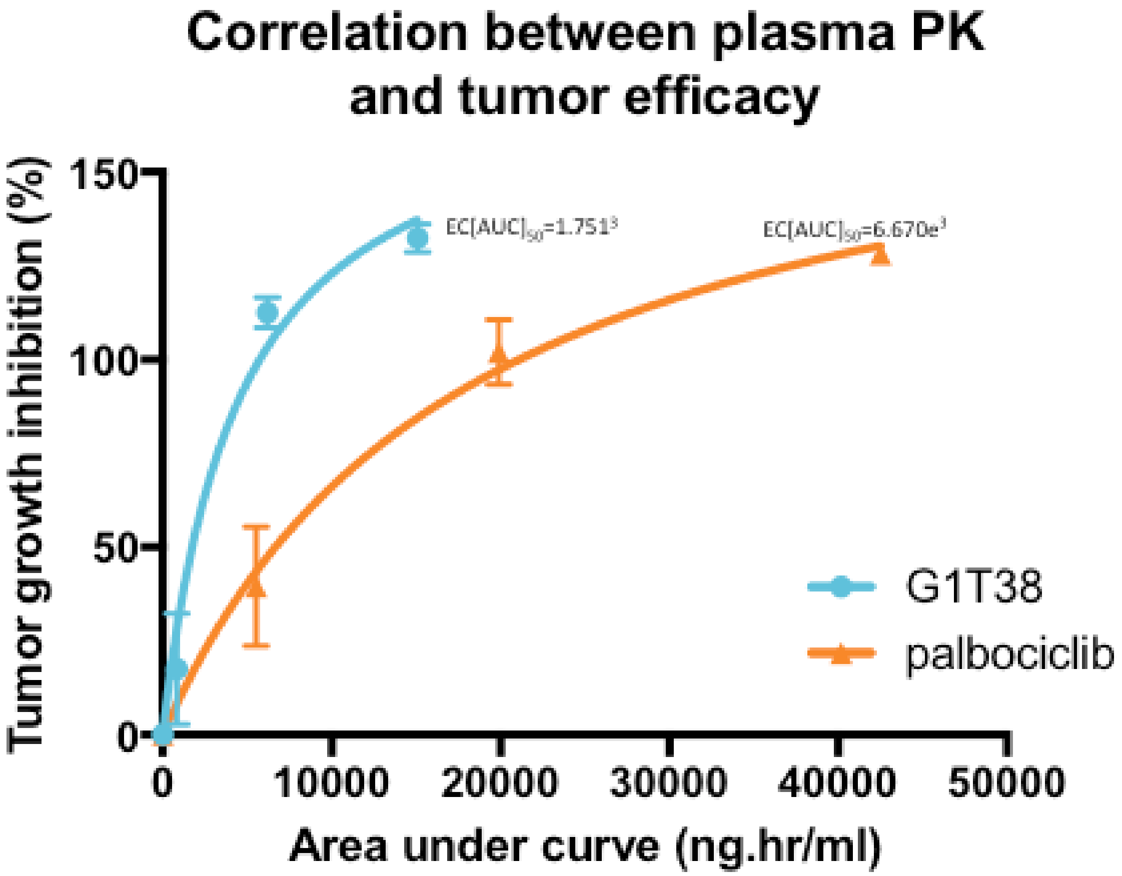

Supplementary Figure 4: Comparison of PK exposure ($AUC_{0\text{-last}}$, single dose) and tumor growth inhibition (MCF7 murine tumors, 28 days of continuous treatment) of palbociclib or G1T38 daily oral treatment as previously described was analyzed using Michaelis-Menten nonlinear regression analysis. Error bars represent SEM.

Supplementary Table 1: G1T38 Kinase Interactions

Target	Kd (nM)
CDK4/ CyclinD1	0.15
CDK4/ CyclinD3	0.05
FLT3 (D835V)	6.5
FLT3 (ITD, D835V)	22
NEK10	0.18
PRKD2	10
PRKD3	5
SLK	11
SNARK	0.69
TTK	4.2
ULK2	5.5

KdELECT® (DiscoveRx) was used as a follow-up to quantify binding affinity of G1T38-kinase interactions identified in the primary screen. Inhibitor binding constants (Kd values) were calculated from duplicate 11-point dose-response curves for targets that elicited >90% inhibition at 100 nM.

Supplementary Table 2: Elimination Half-Lives of G1T38

$T_{1/2}$ (hrs)	Mouse	Rat	Dog	Human
**G1T38**	2.4-5.1	3.1	10.5-14.7	15
**Palbociclib**	2.5-4.2	2.4-4.9	20.7	29

Elimination half-lives ($T_{1/2}$) were determined for G1T38 (in-house results and calculated as part of NCT02821624) and palbociclib [45]. In lower order species, mice and rats, values are similar between both compounds, however in dogs and humans G1T38 these values are approximately one half of palbociclib.

Supplementary Table 3: Accumulation Potential of G1T38 in Rat and Dog Plasma

28-day daily oral treatment			
Rat		Dog	
Dose (mg/kg)	Mean Accumulation Ratio	Dose (mg/kg)	Mean Accumulation Ratio
2.5	0.939	1	1.08
5	0.958	2.5	1.3
15	1.22	5	1.14

Assessment of G1T38 accumulation potential in plasma from 28-day repeat dose experiments in Wistar Han rats and beagle dogs. Across the dose range, mean systemic exposure ($AUC_{0\text{-}24hr}$) to G1T38 after repeated daily oral gavage administration for 28 days did not change meaningfully from that on Day 1. The accumulation ratio (R) was calculated for each animal using the following formula: $R = AUC_{0\text{-}24hr}$ Day 28 $\div$ $AUC_{0\text{-}24hr}$ Day 1.
